# Report of the Diseases of Edinburgh for December, 1807

**Published:** 1808-02

**Authors:** J. Roberton


### *Report of the Diseases of Edinburgh for December,* 1807. By Mr. J. Roberton.

The frost still continues to be very intense, and the winds which accompany it are frequently very high, rendering the cold almost intolerable; even the hardy natives of this country complain most grievously of the severity of the weather. On the 7th of December, at one o'clock A. M. the thermometer in the city stood at 17 degrees below the freezing point; and in the vicinity of Edinburgh it was, at the same time, 20 below the freezing point. Such a degree of cold is not often experienced in this country.

About the middle of the month there was a gradual though very general thaw; and in the course of a few days, the immense quantities of snow which had fallen about the end of last month, were dissolved. During the latter part of the month the weather was, particularly in the day, uncommonly mild. The thermometer now stood, almost constantly, at from 40 to 46. Sometimes, particularly after sun-set, the city was envelloped in the thickest fog that perhaps has ever been seen in this country; nevertheless, the barometer stood almost constantly very high. During the prevalence of easterly winds, we are seldom free from these fogs; but are completely free from them when the wind blows from any other quarter. These fogs are often so very great, as to render the city completely invisible, except a few yards around the spectator; and the chilly sensations which are at all times felt during their continuance, are even, to the most healthy, indescribably unpleasant. Those, however, liable to be affected with chronic disorders, dread their approach; and many, from certain feelings, not of a very agreeable nature, can, several days previous to any apparent change of weather, foretell the particular nature of it with considerable accuracy. Nor is it patients affected with chronic complaints alone, who suffer from such changes of weather; for, owing to this, I believe many of our most acute diseases, if they do not arise from it, are certainly rendered much more severe by it. At these times, therefore, the greatest attention, both by the physician and patient, is absolutely necessary. We often find, that notwithstanding the most unremitting perseverance and care of the medical attendant, sudden changes in the state of the weather have often baffled our every exertion to remove some diseases, which, under different circumstances, were easily easily cured; even some of the complaints of this place have, on such occasions, been reproduced after they had, for several weeks, been removed.

The Inflammatory Diseases, mentioned in last Report, still continue very common, and are, in many instances, suddenly fatal. Others again have been very tedious, notwithstanding every activity of treatment that could be employed. Some of the cases of cynanche tonsillaris, which began last month, still continue with considerable violence; this has been partly owing to the obstinacy of the disease, and partly to the patient's want of attention to himself. The mild weather that occurred about the latter part of the month, induced some of those, who had been confined by this disease, to leave their apartments rather too soon; and many of them, in a convalescent state, were inconsiderately tempted to lay aside some of their additional cloathing. In consequence of these measures, many of them have suffered a relapse, and have been affected more severely than even during the first attack of the disease. Bleeding, locally or generally, blistering, and the liberal use of purgative medicines, have all, in some cases, been found necessary to remove completely such affections.

Inflammation in the Bowels, particularly among children, still continues with considerable severity. Although blistering the abdomen is a very inconvenient application with children, yet, I have found greater benefit from this practice, with the addition of gentle purgatives, than any other recommended by authors.

Pneumonia seems rather beginning to abate in its frequency; still, however, some very obstinate cases, requiring the most active treatment, occur.

Hydrocephalus Internus too has been very common; indeed, from the general inflammatory tendency in the whole system, this might have been expected.*

Catarrh still continues very common among us. The remedies I mentioned in my last Report, seem well calculated to remove it; indeed, much better than any other

N 4 which

* It will seem to some strange, that I have placed this among inflammatory diseases; but by the train of investigation, which I conducted along with Dr. Sanders, during which we discovered the real nature of the disease to be in general of an inflammatory nature, I have no hesitation in stating it as such, and likewise that it is curable by the same means employed for the removal of inflammatory complaints in general. But more of this hereafter.

which I have seen employed, or heard of being employed, by other medical gentlemen.

Chin Cough has considerably abated with us; but in proportion as it abates here, it seems to extend its influence to the surrounding country. In most of the country towns, I believe they seldom or never attempt to remove this disease. Whether this mode, or our plan, of employing six or perhaps eight completely different modes of treatment, is best, I am at a loss to determine. I, however, think, that a total neglect of the disease, in many instances, cannot be worse than some of the plans adopted in our part of the world.

Early in the month, Scarlatina, it is said, made its appearance among us, but it ceased in a very short time. I did not see any cases of it; and I am inclined to think, from the account that has been given me of it, that it has not been scarlatina at all, but the eruptive stage of the measles in some of those patients previously affected with cynanche tonsillaris.

Croup has been observed in a few instances. At one time, this was a very common disease in Edinburgh, but of late years it has almost entirely disappeared. This change, by those who account for every thing, has been attributed to the removal of that mass of filth and nastiness, surrounded by a considerable extent of marshy ground, over the seat of which the North Bridge is built, and which extends along the whole North side of the Castle. It is possible that the removal of this has considerably assisted in the prevention of the disease above alluded to; for, since it took place, we have been very little troubled either with Croup or Intermittent Fever.

I have lately seen a considerable number of Abscesses, in various parts of the body, but more particularly about the great joints. In several instances, both among adults and children, they have also appeared about the angles of the lower jaw. I first observed this affection among children, and suspected it to depend on teething; but although I scarified the gums very liberally, in order to allow the teeth a fair opportunity of coming forward, the process of suppuration did not seem in the least retarded or lessened by it. In addition to this, when I suspected the general inflammatory tendency in the system to be the principal cause, and the teeth to be only partly the cause of this disease, I have employed, without very evident advantage, the strictest antiphlogistic regimen, with the application of leeches and the administration of purgative medicines.

These

These tumors, having often burst externally, have caused much distress to the parents, as well as to the patients themselves, particularly if females, lest, in compliance with the present mode of dress used by the ladies, the scar should give rise to unfair suspicions of the person being affected with scrofula. I think it is a very fair conjecture, that these abscesses have solely been caused by the very great inflammatory diathesis that has of late been so uncommonly prevalent in this country.

A few days ago, I was informed by a friend of mine, who has of late paid some attention to comparative anatomy, that he has found very strong marks of inflammatory action in the viscera of several of the inferior animals that he has examined.

Measles has been the most prevalent disease of this month. It began very early, and rapidly spread over every part of the city and its vicinity. The severity of this disease depends, in general, very much on the time of the year in which the epidemic occurs. In 1799, it made its appearance here during the winter months, and the next time it became prevalent, was in the summer of 1803.—The disease seemed, at both these periods, equally severe, and the treatment adopted in both instances, was nearly the same. Yet in 1799, several hundreds died of it; but in 1803, the number of deaths was very inconsiderable. Undoubtedly, the season of the year in which the epidemic appears, has considerable influence in moderating its violence; particularly when our treatment of the complaint is so discordant and contradictory. I may mention, that it has already proved fatal in several instances. If it immediately succeeds chin cough, it is still more fatal, and, in some instances, where it has been immediately succeeded by diarrhœa, it has almost always terminated fatally. This diarrhœa has been by no means severe, but seems to exhaust the strength of the patient very fast. So far as my opportunities of observation extend, it appears to be irremediable by any means commonly used for the suppression of such complaints. The practice of medical men in this disease is equally discordant with that which I formerly mentioned when speaking of Chin Cough. One recommends blistering on different parts of the body; another disapproves of this practice. One recommends diaphoretics; and in opposition to this, the application of cold air is recommended by others. Purging has likewise been recommended by some, and others will not admit of purging till desquamation of the eruption takes place. Generaland and local bleeding have likewise been recommended, according to the severity of the inflammatory symptoms. So far as I have been able to observe the merits of these different modes of treatment, that which seems to me most successful, is immediately on the first appearance of the disease, to confine the patient to bed with a very moderate share of bed-clothes, in an apartment at about 60° of Farenheit's scale; and at this period, I think it a good general rule, frequently to apply leeches to the temples, and, both before and after desquamation takes place, to use purgatives, as the bowels may seem to require them; and if cough or dyspnœa follows this stage, the repeated application of blisters to the chest, with the use of the pills which I mentioned in my last Report for Chronic Catarrh, must be continued till these symptoms abate.

I cannot close this Report without again remarking, that something ought to be done for the prevention of the Small-pox, either similar to what I formerly hinted, (see Report for October,) or any other plan that may seem more beneficial. In this place they still bear a great proportion among the diseases, which is to be deeply regretted, considering the great mortality that has always attended this disease. We, with uncommon alacrity, issue orders for vessels to be put under quarantine, even if they are supposed to contain diseases which we have reason to believe cannot exist in our climate, and yet tamely allow diseases among ourselves to be often wilfully propagated, which add more to the mortality that prevails in our country, than any other sort of diseases with which we are acquainted.

*No. 4, St. James's Street.*

